# The mediating effect of resilience and COVID-19 anxiety on the relationship between social support and insomnia among healthcare workers: a cross-sectional study

**DOI:** 10.3389/fpsyt.2024.1328226

**Published:** 2024-02-13

**Authors:** Dongmei Zhang, Xiaoping Li, Ming Zhang, Anle Huang, Liu Yang, Congzhi Wang, Ting Yuan, Yunxiao Lei, Haiyang Liu, Ying Hua, Lin Zhang, Jing Zhang

**Affiliations:** ^1^ School of Nursing, Wannan Medical College, Wuhu, Anhui, China; ^2^ School of Innovation and Entrepreneurship, Wannan Medical College, Wuhu, Anhui, China; ^3^ Student Health Center, Wannan Medical College, Wuhu, Anhui, China; ^4^ Nursing Department, The People's Hospital of Yingshang, Yingshang, Anhui, China

**Keywords:** insomnia, COVID-19, healthcare workers, healthcare services, social support, resilience, anxiety

## Abstract

**Background:**

Insomnia in healthcare workers has become a topic of concern in the health system. The high infectivity and longevity of the COVID-19 pandemic have resulted in great pressure and a high incidence of insomnia among healthcare workers. Insomnia among healthcare workers has a negative impact on high-quality healthcare services in addition to their health. Thus, it's necessary to explore insomnia's underlying mechanisms.

**Object:**

The present research's aims were threefold: explored the association between social support, resilience, COVID-19 anxiety, and insomnia among healthcare workers during the pandemic, elucidated the underlying mechanism of insomnia, and offered recommendations for improving the health of these workers.

**Materials and methods:**

A cross-sectional design was adopted. From May 20 to 30, 2022, 1038 healthcare workers were selected to fill out the Oslo 3-item Social Support Scale, the eight-item Athens Insomnia Scale, the Coronavirus Anxiety Scale, and the Brief Resilience Scale. Descriptive statistics and correlations were analyzed by SPSS 25.0. Mediation analysis was conducted by Mplus 8.3 using 5000 bootstrap samples.

**Results:**

Of the participating 1038 healthcare workers, the prevalence of insomnia was 41.62% (432/1038). Significant associations were found involving insomnia, resilience, COVID-19 anxiety, and social support. Insomnia was directly affected by social support. Moreover, three indirect pathways explain how social support affected insomnia: resilience's mediating role, COVID-19 anxiety's mediating role, and the chain-mediation role of resilience and COVID-19 anxiety.

**Conclusion:**

The results validated our hypotheses and supported the opinion of Spielman et al. ‘s three-factor model of insomnia. Social support of healthcare workers has an indirect impact on insomnia in addition to its direct one via independent and chain-mediation effects of resilience and COVID-19 anxiety.

## Introduction

The COVID-19 pandemic spread globally for more than three years from March 2020 to May 2023 (1, 2). To curb its spread, the Chinese government formally declared that it would implement the ‘‘dynamic zero-COVID policy” in December 2021 (3). When one case takes place, the policy mandates that prompt and appropriate action should be taken. From March 2022 to January 2023, the epidemic resurfaced extensively and regularly (4). Omicron, the most prevalent strain, was extremely contagious and caused the studied wave of the pandemic (5). With the high population density in China, the outbreak was quick and widespread, albeit high-risk areas in strict lockdown (6).

Accordingly, the medical system was directly under huge pressure and risk. Healthcare workers, who comprised the primary force behind the pandemic response, were under immense physical and psychological strain due to the rise of COVID-19 cases. They were overburdened with the daily task of screening every population in high-risk areas, treating COVID-19 patients, being at high risk of infection, and uncertainty of the epidemic (4, 7). All of these stressors made them vulnerable to mental problems such as insomnia and anxiety (8–10). Healthcare workers had greater prevalences of anxiety and insomnia than the overall population, because of the high demands and great stress during the pandemic (11). Yuan et al. (11) have validated that insomnia symptoms and anxiety among healthcare workers were 39.3% and 35.9%, respectively. As time progressed, the prevalence of psychological problems such as insomnia rose during the COVID-19 pandemic among healthcare workers (11).

Insomnia refers to dissatisfaction with sleep either qualitatively or quantitatively, including problems falling asleep, staying asleep, waking up early in the morning, and not being able to go back to sleep (12). Insomnia relates to a number of illnesses, such as hypertension, diabetes, stroke, and chronic kidney illness (12). Also, insomnia relates to accidents on the road, workplace, and home (13, 14). As a significant predictor for anxiety and depression, insomnia has bidirectional effects on anxiety and depression (15, 16). For healthcare workers, insomnia contributes to exhaustion (17). Exhaustion can easily lead to malpractice because it badly affects healthcare workers ability to tackle tasks involving attention, cognition, and memory (18). In all probability, insomnia is a group phenomenon during the pandemic (19). Insomnia among healthcare workers harms high-quality healthcare services in addition to their health. Therefore, it is imperative to put more emphasis on insomnia and influencing mechanisms among healthcare workers.

Spielman et al. 's three-factor model of insomnia (20) provides a theoretical framework for this study. In the model, predisposing, precipitating, and perpetuating factors can contribute to insomnia. Firstly, predisposing factors include demographic, biological, psychological, and social characteristics. The tendency to worry excessively and stress at work belong to predisposing factors of insomnia. An individual's capacity to adapt to and flourish in challenging circumstances is known as resilience (21, 22). A prior study (23) validated that insomnia is related to a lower level of resilience. In this sense, a lower level of resilience can be seen as a predisposing factor for insomnia. Secondly, precipitating factors generally comprise stressful events or medical conditions that may disrupt sleep. As mentioned above, the pandemic has contributed to anxiety and insomnia among healthcare workers (8–11). Thirdly, perpetuating factors generally include behavioral or cognitive changes provoked by acute insomnia. Cognitive models of insomnia emphasize the role of anxiety in the onset and persistence of insomnia (24). It has been reported that insomnia relates to anxiety during COVID-19 (7, 25).

Social support refers to existing or available social resources when needed (26). The four types of social support are instrumental support, emotional support, informational support, and appraisal support (27). Resilience refers to positive qualities, such as the ability to make good use of social support, self-efficacy, and growth from setbacks (28). Social support may relieve the adverse effects on insomnia and anxiety (29). Moreover, social support influences pressure regulation through affecting resilience according to Kumpfer's theory (30). A prior study (31) has suggested that resilience can relieve COVID-19 anxiety.

In sum, healthcare workers encounter multiple pressures during the pandemic, which may contribute to anxious feelings or anxiety. Some maladjusted healthcare workers may further develop insomnia. Not only does social support directly affect insomnia, but it may also affect an individuals insomnia by affecting their resilience and anxiety.

Insomnia in healthcare workers has become a topic of concern in the health system. However, insomnia and its underlying mechanisms have not been explored in terms of social support, resilience, and anxiety among healthcare workers during the pandemic. Consequently, the present research's aims were threefold: explored the association between social support, resilience, COVID-19 anxiety, and insomnia among healthcare workers during the pandemic, elucidated the underlying mechanism of insomnia, and offered recommendations for improving the health of these workers.

**Figure 1:**
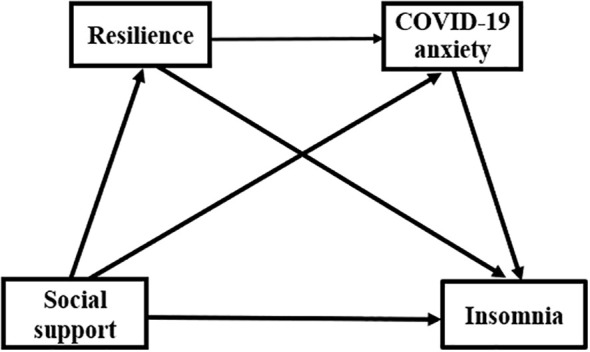
Hypothesized relationship model of variants.

Four hypotheses were postulated. Hypothesis 1: social support and insomnia are directly correlated. Hypothesis 2: resilience is a mediator of social support and insomnia. Hypothesis 3: COVID-19 anxiety is a mediator of social support and insomnia. Hypothesis 4: resilience and COVID-19 anxiety mediate the relationship in a sequential manner (**Figure 1**).

## Materials and methods

### Design

A cross-sectional design was to explore the relationship among insomnia, resilience, COVID-19 anxiety, and social support in healthcare workers.

### Participants

The participating healthcare workers were recruited from four hospitals in Fuyang City, where the extremely infectious Omicron outbreak swept over the whole region. Since there are 22 items over the four scales, a sample size of at least 242 was determined using Kendall's criterion, which calls for multiplying the total number of items by at least ten times and adding no less than 10% (32). The inclusion criteria are (1): a full-time healthcare worker in the medical facility, and (2) volunteer participation in this investigation. The healthcare workers were invited via WeChat, a popular Chinese social App. The survey materials were distributed using Questionnaire Star. Questionnaire Star was employed because it is a user-friendly online service platform for questionnaire surveys and social distancing was restricted during the pandemic (33, 34). Prior to the data collection, each participant provided their informed consent via Questionnaire Star. In the informed consent part, each participant was informed of the study aims and their anonymity. The participants could fill in questionnaires only when “agreement” was chosen. Then, they could click on the choice in the filling interface. The returns included 1097 questionnaires received from May 20 to May 30, 2022, of which 1038 (95.14%) were eligible. 59 questionnaires were ineligible because the answers were considered insincere (such as the first option offered chosen consistently). The Ethical Review Committee of the School of Nursing, Wannan Medical College gave its approval to this study (20220004).

### Measures

#### Sociodemographic information

With reference to previous studies (35–38), sociodemographic data in the study included sex, age, occupation, professional titles, and whether they were frontline healthcare workers.

#### The eight-item Athens insomnia scale

Developed by Soldatos et al. (39), the self-reporting eight-item AIS-8 scores items from zero (no) to three (very serious). Manzar et al. (39) have validated that the AIS-8 possesses excellent validity and reliability. Niu et al. (40) validated the Chinese version has acceptable reliability. The Cronbach's α coefficient of AIS-8 was 0.880 in the present research.

#### Coronavirus anxiety scale

The 5-item CAS was originally developed by Lee et al. (35) and scores items from zero to four. The Chinese version of CAS has been validated with good psychometric properties (41). Anxiety related to COVID-19 increases with greater CAS total scores. The Cronbach's α coefficient was 0.897 in this study.

#### Brief resilience scale

The BRS possesses 6 items. Each item on BRS is scored from one to five (42). Resilience increases with greater BRS total scores. A prior study reported by Kim et al. (42) has validated that the BRS possesses good psychometric properties. A Chinese study (43) has also demonstrated that the BRS demonstrates good reliability and validity. In the present research, its Cronbach's α coefficient was 0.715.

#### Oslo 3-item social support scale

OSS-3 was employed to evaluate healthcare workers perceived social support levels. Notwithstanding only containing three items, OSS-3 owns good psychometric properties (44). The total scores of OSS-3 range from 3 to 14. In the current research, its Cronbach's α coefficient was 0.669.

### Data analysis

Descriptive statistics and correlations were analyzed by SPSS 25.0. SPSS 25.0 is one of the most widely used statistical analysis software (32). It not only possesses powerful statistical functions but also an easy-to-use interface. We employed the independent samples *t*-test for the comparison of variables (sex, occupation, insomnia or not, and frontline healthcare workers or not). A one-way analysis of variance was employed to measure whether there is a difference between professional titles. Pearson correlation analysis was adopted to explore the associations among variables. Regression analysis was conducted by model 6 of the SPSS PROCESS macro (45). In the meanwhile, the influence of participants characteristics (sex, age, occupation, professional titles, and frontline healthcare workers or not) was adjusted for. Finally, mediation analysis was conducted by Mplus 8.3 using 5000 bootstrap samples. Mplus 8.3 is a flexible statistical modeling program. It not only possesses graphical displays of data and analysis results, but also a user-friendly interface (46). The mediating model was assessed by fit indexes, with the standard values as chi-square/degree of freedom (*χ*2/*df*)< 5, comparative fit index (CFI)< 0.90, Tucker-Lewis index (TLI)< 0.90, standardized root mean square residual (SRMR)< 0.05, and root-mean-square error of approximation (RMSEA)< 0.08 (47).

## Results

### Participants characteristics

The age range was 18 - 68 years old. As shown in **Table 1**, most of them were females (800, 77.07%), nurses (698, 67.24%), junior professionals (749, 72.16%), and frontline healthcare workers (807, 77.75%). Of the 1038 healthcare workers, 432 were screened out for insomnia, with a prevalence of 41.62%. Moreover, respondents with significantly higher levels of insomnia were found among the female (*t* = -3.032, *P* = 0.002), the intermediate (*F* = 4.621, *P* = 0.010), and frontline healthcare workers (*t* = 2.697, *P* = 0.007).

**Table 1:** Characteristics of participants and insomnia (*N* =1038).

Variable	Groups	*N* (%)	$\bar{X}$ ± *S*	*t/F*	*P*
Sex	Male	238 (22.93)	5.98 **±** 4.44	-3.032	0.002
	Female	800 (77.07)	6.94 ± 4.25		
Occupation	Nurse	698 (67.24)	6.89 ± 4.27	1.780	0.075
	Doctor	340 (32.76)	6.38 ± 4.38		
Professional title	Junior	749 (72.16)	6.58 ± 4.31	4.621	0.010
	Intermediate	226 (21.77)	7.42 ± 4.41		
	Senior	63 (6.07)	5.89 ± 3.72		
Frontline workers	No	231 (22.25)	6.04 ± 3.96	2.697	0.007
	Yes	807 (77.75)	6.91 ± 4.39		
Insomnia	No	606 (58.38)	3.77 ± 2.21	42.730	<0.001
	Yes	432 (41.62)	10.87 ± 2.91		

### Relationships of variables

**Table 2** showed the significant correlations between the four variables. Specifically speaking, any one variant was significantly linked to the other three variants (*P* < 0.001).

**Table 2:** Descriptive statistics and correlations among variants (*N* =1038).

Variable	$\bar{X}$ ± *S*	Social support	Resilience	COVID-19 anxiety	Insomnia
Social support	10.04 ± 2.03	1.000			
Resilience	20.07 ± 4.23	0.392^***^	1.000		
COVID-19 anxiety	5.91 ± 2.42	-0.197^***^	-0.268^***^	1.000	
Insomnia	6.72 ± 4.31	-0.366^***^	-0.426^***^	0.386^***^	1.000

^***^P <0.001.

### Common method bias test

Prior to analyzing the data of the structural equation model, the common method bias test was conducted using the Harman single factor test. There was no obvious multicollinearity since the variance presented by the first factor was 30.788% (48).

### Regression analysis

Social support was a positive predictor of resilience (β = 0.794, *P* < 0.001). Also, it was a negative predictor of COVID-19 anxiety (β = -0.130, *P* < 0.001) and insomnia (β = -0.428, *P* < 0.001). Resilience was a negative predictor of COVID-19 anxiety (β = -0.131, *P* < 0.001) and insomnia (β = -0.281, *P* < 0.001). COVID-19 anxiety was a positive predictor of insomnia (β = 0.472, *P* < 0.001) (**Table 3**).

**Table 3:** Results of regression analysis.

Variable	Resilience			COVID-19 anxiety			Insomnia		
	β	Se	*t*	β	Se	*t*	β	Se	*t*
Sex	-1.244	0.373	-3.338^***^	-0.151	0.226	-0.669	0.470	-0.349	1.346
Age	-0.001	0.021	-0.040	0.015	0.012	1.218	0.029	0.019	1.537
Occupation	0.115	0.333	0.343	-0.162	0.201	-0.804	-0.096	0.311	0.308
Professional title	0.238	0.265	0.899	0.022	0.160	0.137	-0.075	0.247	-0.302
Frontline worker	-0.324	0.308	-1.052	-0.015	0.186	-0.081	-0.844	0.288	-2.935^**^
Social support	0.794	0.060	13.336^***^	-0.130	0.039	-3.346^***^	-0.428	0.060	-7.074^***^
Resilience				-0.131	0.019	-6.936^***^	-0.281	0.030	-9.456 ^***^
COVID-19 anxiety							0.472	0.048	9.781^***^
*F*		31.484			12.407			51.662	
*P*		<0.001			<0.001			<0.001	
*R^2^*		0.177			0.088			0.312	

^**^P < 0.01, ^***^P < 0.001.

### Mediating analysis

We employed the mediating analysis to elucidate the underlying mechanism of insomnia and enhance the theoretical depth (32). The mediating model possessed ideal fit indexes with *χ*2/*df* = 2.030, CFI = 0.994, TLI = 0.986, SRMR = 0.018, RMSEA = 0.031 (**Table 4**) (47).

**Table 4:** Evaluating the fit of the mediating model.

aModel	*χ*2/*df*	CFI	TLI	SRMR	RMSEA
Mediating model	2.030	0.994	0.986	0.018	0.031
Standard value	<5.000	>0.900	>0.900	<0.500	<0.080

**Figure 2:**
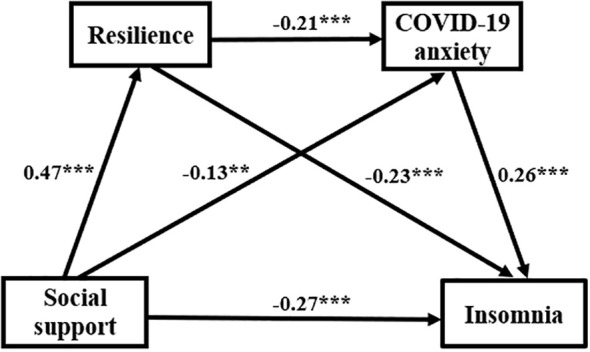
Pathway displaying resilience and COVID-19 anxiety in the connection between social support and insomnia. ^**^ *P <*0.01, ^***^ *P <*0.001.

As for mediating effects, we mainly employed effect size, 95% confidence interval (CI), and relative effect. **Table 5** and **Figure 2** revealed that insomnia was directly negatively affected by social support (β = -0.268; 95% CI: -0.351 - -0.184), corresponding to 61.61% of the whole effect, which validated hypothesis 1. There were three indirect pathways between social support and insomnia. First, resilience's mediating role (β = -0.108, 95% CI: -0.145 - -0.075) corresponded to 24.83% of the whole effect, which validated hypothesis 2. Second, COVID-19 anxiety's mediating role (β = -0.034, 95% CI: -0.059 - -0.013) corresponded to 7.82% of the whole effect, which validated hypothesis 3. Third, the chain-mediation role of them (β = -0.025, 95% CI: -0.041 - -0.014) corresponded to 5.75% of the whole effect, which validated hypothesis 4.

**Table 5:** Mediating effects of social support and insomnia.

	Paths	Effect	Boot SE	95% CI	Relative effect
Direct effect	Social support→Insomnia	-0.268	0.043	-0.351 - -0.184	61.61%
Indirect effect	Social support→Resilience→Insomnia	-0.108	0.018	-0.145 - -0.075	24.83%
	Social support→COVID-19 anxiety →Insomnia	-0.034	0.012	-0.059 - -0.013	7.82%
	Social support→Resilience→COVID-19 anxiety →Insomnia	-0.025	0.007	-0.041 - -0.014	5.75%
Total indirect effect		-0.167	0.021	-0.211~-0.130	38.39%
Total effect		-0.435	0.036	-0.503~ -0.361	100.00%

## Discussion

As the health status of healthcare workers adversely affects high-quality medical services, it is vitally important to elucidate the underlying mechanism of insomnia and offer practical guidance to healthcare workers mental health. The current study analyzed insomnia among healthcare workers and the relationships between social support, resilience, COVID-19 anxiety, and insomnia. The results validated our hypotheses and supported the opinion of Spielman et al.'s three-factor model of insomnia, offering a theoretical foundation for improving the health of healthcare workers.

### Characteristics of insomnia in healthcare workers

The current study revealed that 41.62% of healthcare workers had insomnia, which was slightly higher than Yuan et al.'s research (11). This may be due to the relatively large proportion of frontline healthcare workers in our research. In line with prior research (10, 49), the present study showed that frontline healthcare workers had a notably greater prevalence of insomnia than others. In comparison with non-frontline colleagues, frontline healthcare workers experienced more stress which contributed to insomnia. Specifically, frontline healthcare workers expressed much fear and anxiety because of their direct contact with COVID-19 patients, worry about being infected, and heavy workload (50). Separation from their family reduced family support. These factors lead to the high prevalence of insomnia in frontline healthcare workers. This research validated that insomnia was more common in females than in males, which was in line with previous studies (11, 51). Compared with males, females had lower resilience and higher perceived stress (52). Additionally, females were more emotional than males and tended to express their concerns to their family and friends (53), while the isolation from family and high workload impacted this need for emotional support. Intermediate healthcare workers were more likely than junior and senior colleagues to experience insomnia. This is probably because the majority of intermediate healthcare workers were also parents whose children needed to be cared for. They played an important role both in the hospital and at home. As a result, their anxiousness naturally increased (54), which led to insomnia directly. Significantly, more attention should be paid to female, frontline, and intermediate grade healthcare workers in the prevention and treatment of insomnia.

### Insomnia was directly negatively affected by social support

The current research revealed that insomnia was directly negatively affected by social support, which agreed with prior research (10, 55). Job demands-control-support (JDCS) model (56) proposes that high demand and low work control can contribute to stress and health problems, while social support can buffer the impact of stress. Suffering multiple stresses and low work control during the pandemic, healthcare workers urgently needed social support. People with more social support would have more resources to prevent insomnia and a greater possibility to reengage with their lives following a stressful event (57). For example, empathy can directly reduce arousal because it enables people who are experiencing pain to relate their negative emotions (29).

### Social support and insomnia were mediated by resilience and COVID-19 anxiety, respectively

Moreover, the current research validated that social support and insomnia were mediated by resilience and COVID-19 anxiety, respectively. These indicated that resilience strengthened the beneficial impact of social support on insomnia, while COVID-19 anxiety diminished the effect.

The result, resilience acting as a mediator between social support and insomnia, supported the opinion of Kumpfer's theory. The theory (30) proposes that the processes of human interaction with the environment are self-integration in favor of resilient reintegration. As an external environmental and protective factor, social support contributes to positive responding with resilient reintegration. Fu et al. (18) have demonstrated that resilience is vital for suppressing insomnia. During the new wave of the pandemic, healthcare workers faced huge pressure (7). In this case, healthcare workers with higher resilience were more likely to take advantage of social support, which may have prevented or permitted them quickly to recover from insomnia (18, 58). To prevent and reduce insomnia, it is necessary to improve resilience and provide effective social support for healthcare workers.

COVID-19 anxiety acting as a mediating role between social support and insomnia, lent credence to the Conservation of Resources theory (COR). COR theory (59) indicates people experience stress and anxiety, when they are in danger of losing resources or have lost resources. During the pandemic, there was a clear correlation between the loss of resources and psychological distress (60). During the lockdown, healthcare workers were exhausted and threatened by infection. Correspondingly, anxiety was triggered easily among healthcare workers (7, 61). Insomnia often occurs concurrently with or following the onset of anxiety (62), because they have a common pathophysiological mechanism (63). Li et al.'s study has shown there is a positive correlation between the regional homogeneity values of the right supplementary motor area and the anxiety score (64). In the anxious individual, reduced sleep is related to increased connectivity between the hippocampus and the insula as the individual with insomnia often worries too much about sleep and the hazards of insomnia (65). This anxiety leads to selective attention toward adverse cues related to sleep. Ultimately, excessive and escalating anxiety may lead to insomnia (24). So, anxiety plays a role in both causing and sustaining insomnia. According to COR, supplementing of resources is vitally important and valuable following resource loss, which can relieve the tension and pressure of the individual who already has few resources (66). Social support is essential for supplementing resources (32). Social support can protect healthcare workers from insomnia by reducing anxiety. Specifically, instrumental support is able to ease the burdens as a result of COVID-19 directly and thus lower anxiety. Emotional support may enhance people's feeling of security and belonging, which contributes to decreasing people's arousal and misery. Consequently, social support, which reduces coronavirus anxiety, could be a benefit for preventing insomnia in healthcare workers. It suggests that social support is a key point when designing interventions to prevent and cure insomnia among healthcare workers.

### Resilience and COVID-19 anxiety acted as a chain mediator between social support and insomnia

This study also showed that resilience and COVID-19 anxiety acted as a chain mediator. It indicated that social support may lower insomnia via affecting resilience and COVID-19 anxiety by improving resilience (27). One person who possesses strong resilience is more likely to employ positive coping measures (67, 68). Social support reduces the high stress on healthcare workers and is beneficial for improving their resilience. Resilience such as positive coping and self-efficiency is beneficial for boosting capacity for recovering from stress. Prior studies have demonstrated that resilience contributed to decrease anxiety during the COVID-19 pandemic (31, 68). When facing high demands and professional risk during the pandemic, healthcare workers with high resilience would feel lower anxiety than the others because of their positive adjustments. A prior study reported by Tang et al. (7) has also demonstrated anxiety is an important factor in causing insomnia of healthcare workers. Thus, social support can affect insomnia among healthcare workers by the chain mediator.

Correspondingly, in terms of hospitals, online and offline measures should be taken to prevent and intervene in healthcare workers insomnia and anxiety during the pandemic. Effective measures such as CoviPsyHUS (a system of preventing and caring for mental health) (69), Stress First Aid (70), the Resilience Coaching program (71), and the Resilience-Building App (72) may be taken. In the long run, enhancing healthcare workers resilience should be incorporated into hospitals continuing education measures. Additionally, it is critical to assist healthcare workers in viewing social support as a resource and taking full advantage of it.

### Limitations and strengths

The cross-sectional design restricted the exploration of causation among the variables. Consequently, a longitudinal design ought to be carried out to validate these findings. Additionally, the participants were healthcare workers recruited from four hospitals. Given that, expanding the sample distribution is essential. Irrespective of these limitations, this study investigated insomnia and influencing mechanisms among healthcare workers during the pandemic and city lockdown. Moreover, the findings can provide the theoretical basis for enhancing healthcare workers health.

## Conclusion

Healthcare workers were apt to be troubled by insomnia during the pandemic and city lockdown. The insomnia of healthcare workers is directly related to healthcare workers health and high-quality medical services. Given that, insomnia deserves particular attention among healthcare workers. Social support of healthcare workers not only influences insomnia directly, but also indirectly by means of the independent and serial mediator of resilience and COVID-19 anxiety. So, the prevention and treatment of insomnia in healthcare workers should be carefully considered. To lessen insomnia, combined tactics aimed at improving social support, boosting resilience, and lowering COVID-19 anxiety are required.

## Data availability statement

The raw data supporting the conclusions of this article will be made available by the authors, without undue reservation.

## Ethics statement

The studies involving humans were approved by the ethical committee of the College of Nursing of Wannan Medical College. The studies were conducted in accordance with the local legislation and institutional requirements. The participants provided their written informed consent to participate in this study.

## Author contributions

DZ: Formal analysis, Funding acquisition, Investigation, Methodology, Project administration, Writing – original draft, Writing – review & editing. XL: Writing – review & editing. MZ: Writing – review & editing. AH: Writing – review & editing. LY: Writing – review & editing. CW: Writing – review & editing. TY: Writing – review & editing. YL: Writing – review & editing. HL: Writing – review & editing. YH: Writing – review & editing. LZ: Data curation, Methodology, Writing – review & editing. JZ: Investigation, Writing – review & editing.
